# Theoretical and computational study of benzenium and toluenium isomers

**DOI:** 10.3389/fchem.2023.1253599

**Published:** 2023-11-01

**Authors:** Falonne C. Moumbogno Tchodimo, Walter C. Ermler

**Affiliations:** Department of Chemistry, The University of Texas at San Antonio, San Antonio, TX, United States

**Keywords:** benzenium, toluenium, proton affinity, hyperconjugation, DFT

## Abstract

Four methods of computational quantum chemistry are used in a study of hyperconjugation in protonated aromatic molecules. Benzene, benzenium, toluene, and four isomeric forms of toluenium are examined using the self-consistent field level of theory followed by configuration interaction and coupled cluster calculations, as well as density functional theory. Results for proton affinities, geometric parameters, atomic populations, dipole moments, and polarizabilities are reported. The calculated results are in good agreement with previous computational studies and with experimental data. The presence of hyperconjugation is evident from the shortened carbon–carbon bond lengths in the aromatic ring and concomitant changes in dipole moments and polarizabilities. The proton affinities of benzene and toluene compare well with experimental values. The examination of all of the toluenium isomers reveals that the position of the methyl group has a minor impact on the strength of hyperconjugation, although the most stable isomer is found to be the para form. Mulliken population analyses indicate that the addition of a proton contributes to aromatic hyperconjugation and increases the strength of π-bonds at the expense of σ-bonds.

## 1 Introduction

The Baker–Nathan effect was discussed initially in 1935 (Baker and Nathan, 1935). It is rooted in the presence of additional resonance structures in certain molecules and, in general, is described as an electron density delocalization from a σ-bond to a p-orbital or a π-bond. In a series of seminal articles, Mulliken extended and generalized the Baker–Nathan concept and coined the term *hyperconjugation* (hereafter denoted as HC) (Mulliken, 1939; Rieke et al., 1941). The importance of delocalizing interactions involving σ-bonds has been examined theoretically and computationally (Reed et al., 1988). It has been reported that HC can influence conformational equilibria (Romers et al., 1969; Zefirov and Schechtman, 1971), modify reactivity (Baddeley, 1973; Chang et al., 1987), and determine selectivity (Beckwith and Duggan, 1998). Furthermore, it is has been established that the presence of HC is mainly responsible for the stability of the more highly substituted alkenes (Fox and Whitesell, 2004). The anomeric effect in the reactivity of α-haloglycine esters with various nucleophiles has been attributed to HC, leading to enhanced halogen nucleofugality which facilitates halogen abstraction by hydrogen-bond donor catalysts (Samanta and Stéphane, 2019). For example, the presence of HC has been attributed to improved catalysis in a pyridoxal 5′-phosphate-dependent enzyme (Dajnowicz et al., 2018), as well as being an important factor in stabilizing certain excited, radical, and ionic species (Muller and Mulliken, 1958; Lambert et al., 1987; Raabe et al., 1996). Davies maintained that HC activation in the presence of Sn–C bonds can accelerate a reaction rate by more than a factor of 10^14^ (Davies, 1999). In organometallic compounds, the hyperconjugative β-proton coupling of an H–C σ-bond in a cyclic radical can be enhanced or decreased according to the symmetry of the π-orbital with which it hyperconjugates according to the so-called Whiffen effect (Davies, 1999). Ermler et al. (1976) carried out accurate *ab initio* self-consistent field (SCF) calculations to examine the role of HC in the benzenium and p-toluenium cations (Ermler and Mulliken, 1978). A recent study that examines the phenomenon of HC in aromatic systems is consistent with their conclusions (Xiaojuan et al., 2020).

## 2 Calculations

**FIGURE 1:**
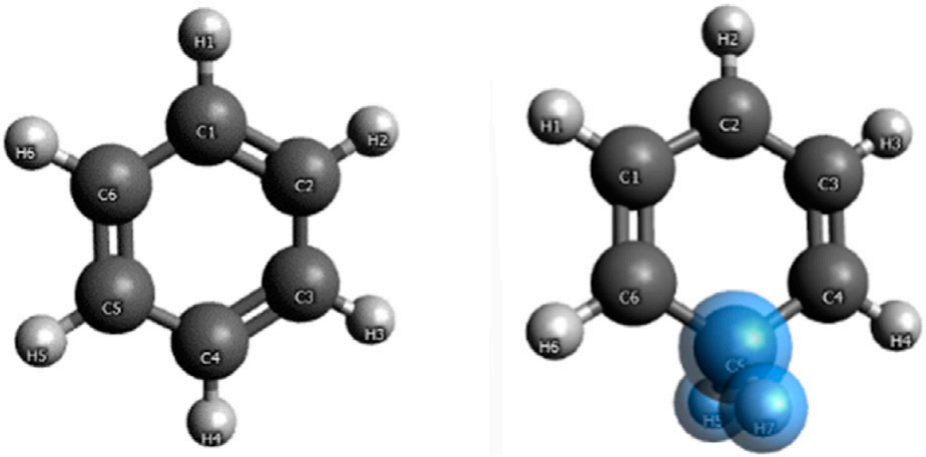
Benzene and benzenium.

**FIGURE 2:**
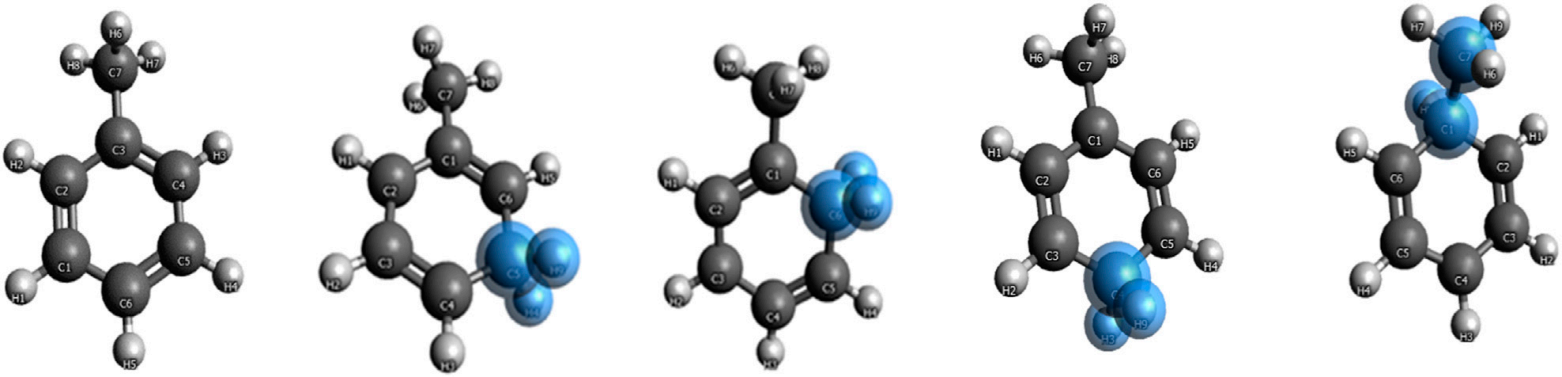
Toluene and meta-, ortho-, para-, and ipso-toluenium.

Benzene and benzenium are shown in Figure 1, and toluene and the four isomeric forms of toulenium are shown in Figure 2. The goal of the present study is to identify and codify the presence of HC in these molecules through a set of high-level calculations of energies, bond lengths, bond angles, electric dipole polarizabilities, and Mulliken populations.

The calculations reported in this study were accomplished using the Gaussian software suite (Frisch et al., 2016). SCF calculations were carried out followed by single plus double excitation configuration interaction (CISD) and coupled cluster with single and double and perturbative triple excitation (CCSD(T)) calculations. All were conducted using 6-311G** basis sets of contracted Gaussian-type functions (Frisch et al., 2016). In addition, density functional theory (DFT) calculations using B3LYP exchange correlation functionals and 6-311G** basis sets were carried out. At each level of theory, a full geometry optimization was accomplished. The proton affinities of benzene and each toluene isomers were calculated at each level of theory. At each level of theory the proton affinity is defined as the difference between the total energy of the neutral species at its calculated equilibrium geometry less than that of the protonated species at its calculated equilibrium geometry. Finally, dipole moments, defined as expectation values of the dipole moment operator, and dipole polarizabilities, defined as the change in the dipole moment of a molecule when in a static electric field, are reported at all levels of theory (Frisch et al., 2016).

Initially, single-point energy SCF calculations were carried out to determine total energies, molecular orbital (MO) energies, Mulliken populations, and electric dipole polarizabilities. Results for the benzene systems and the toluene systems are compared to those obtained by Ermler et al. (1976) and Ermler and Mulliken (1978), respectively. These preliminary SCF calculations were followed by CISD and CCSD(T) calculations for each of these molecules, and the aforementioned properties were calculated. Additionally, the ortho, meta, and ipso forms of toluenium were also studied at the stated levels of theory. Figure 2 shows the position of the methyl group in the four isomers of protonated toluene. Datasets for the ortho, meta, para, and ipso forms of the toluenium molecules were constructed, and geometry optimizations were completed yielding equilibrium bond lengths and bond angles and the corresponding proton affinities. These structures were then used to calculate electric dipole polarizabilities and populations of each molecule.

## 3 Results and discussion

Table 1 compares the present results for benzene and benzenium with those of [17]. The results for toluene and toluenium are compared with those of [18] in Table 2.

**TABLE 1:** Calculated total SCF energy, HOMO energy, and proton affinity for benzene and benzenium compared to the work of Ermler et al. (1976) (a.u.).

	Benzene		Benzenium	
Source	Ermler et al.	Calculated	Ermler et al.	Calculated
Total energy (a.u.)	−230.7771	−230.7540	−231.0784	−231.0635
HOMO energy (a.u.)	−0.337	−0.336	−0.546	−0.561
Proton affinity (kcal/mol)	189.1	194.2	-	-

**TABLE 2:** Calculated total SCF energy, HOMO energy, and proton affinity for toluene and p-toluenium compared to the work of Ermler and Mulliken (1978) (a.u.).

	Toluene		p-Toluenium		
Source	Ermler et al*.*	Calculated	Ermler et al.	Calculated	
Total energy (a.u.)	−269.8245	−269.8000	−270.1399	−270.1243	
HOMO energy (a.u.)	−0.323	−0.324	−0.612	−0.554	
Dipole moment (D)	−0.328	−0.336	-		
Proton affinity (kcal/mol)	197.9	203.5	-		-

The calculated results are in good agreement with those cited in both earlier studies, with differences attributable to the use of the somewhat smaller 6-311G** basis set in the current calculations compared to the σ/π-optimized polarized double zeta basis sets used in the work of Ermler et al. (1976) and Ermler and Mulliken (1978). The main differences in total energies are due to a more rigorous representation of the carbon 1s core electrons by 9 s-type GTOs *versus* the standard Gaussian 6 s-type GTOs in the 6-31G** basis sets. These core energy differences do not appreciably impact the quality of the MOs that describe the C valence electrons. The calculated highest occupied molecular orbital (HOMO) energy values in Table 1 and Table 2 are in excellent agreement with the work of Ermler et al. (1976) and Ermler and Mulliken (1978). Results for HOMO energies (from natural orbitals) and for proton affinities for the CISD calculations using the same 6-311G** basis set are shown in Table 3. It is noteworthy that the CISD values for the proton affinities for benzenium and toluenium from the present calculations are in close agreement with the work of Ermler et al. (1976) and Ermler and Mulliken (1978) and with the experimentally determined (NIST Chemistry WebBook) values of 178.4 and 187.0 kcal/mol, respectively. The calculated dipole moment of toluene agrees well with the experimentally determined (NIST Chemistry WebBook) value of 0.36 D.

**TABLE 3:** CISD and CCSD(T) results for benzene and benzenium.

Energy (a.u.)	Benzene	Benzenium
CISD	−231.4517	−231.7553
CCSD(T)	−231.5824	−231.8831
Proton affinity (kcal/mol) observed	178.4	-
CISD	190.5	-
CCSD(T)	188.7	-

**FIGURE 3:**
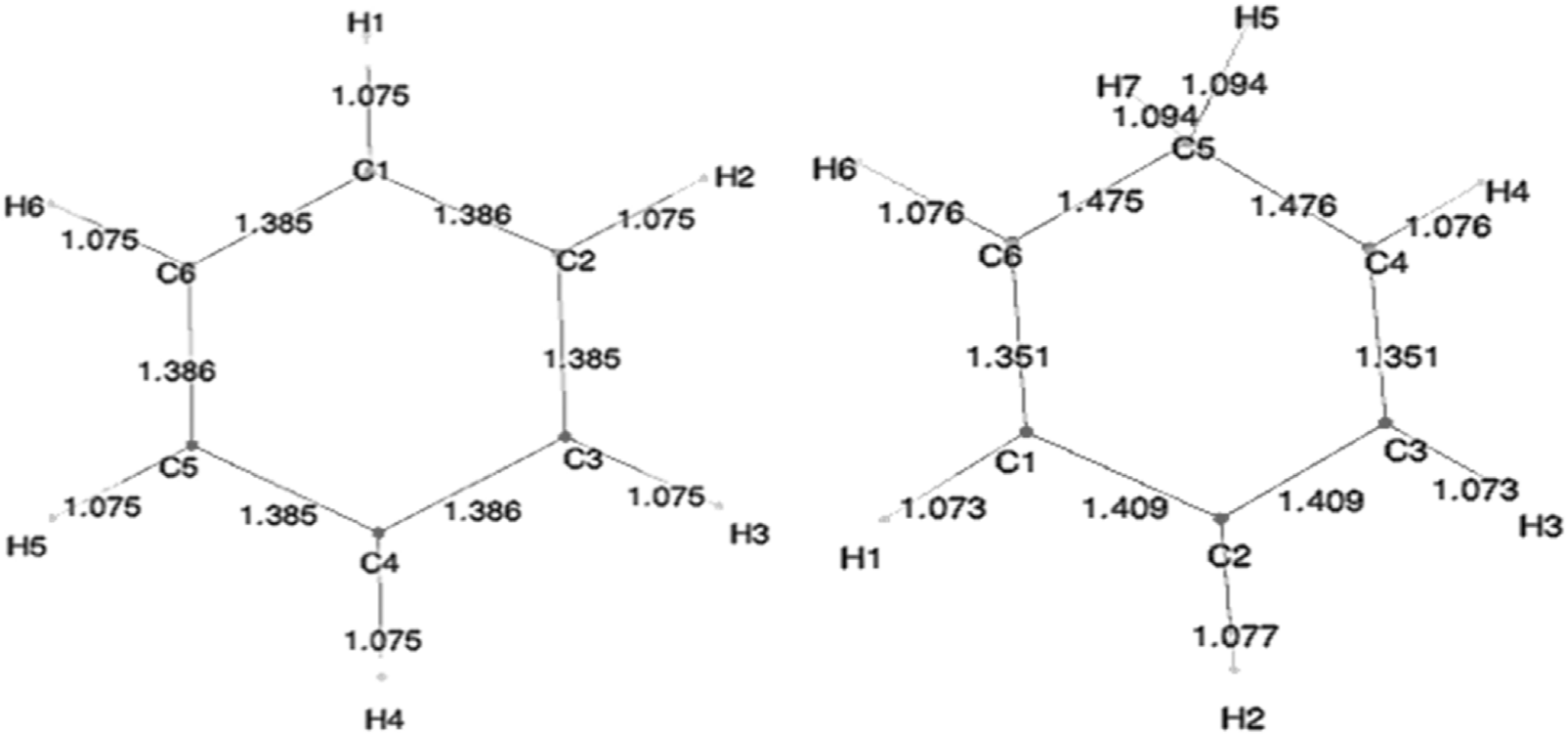
SCF-optimized bond lengths of benzene and benzenium (Å).

**FIGURE 4:**
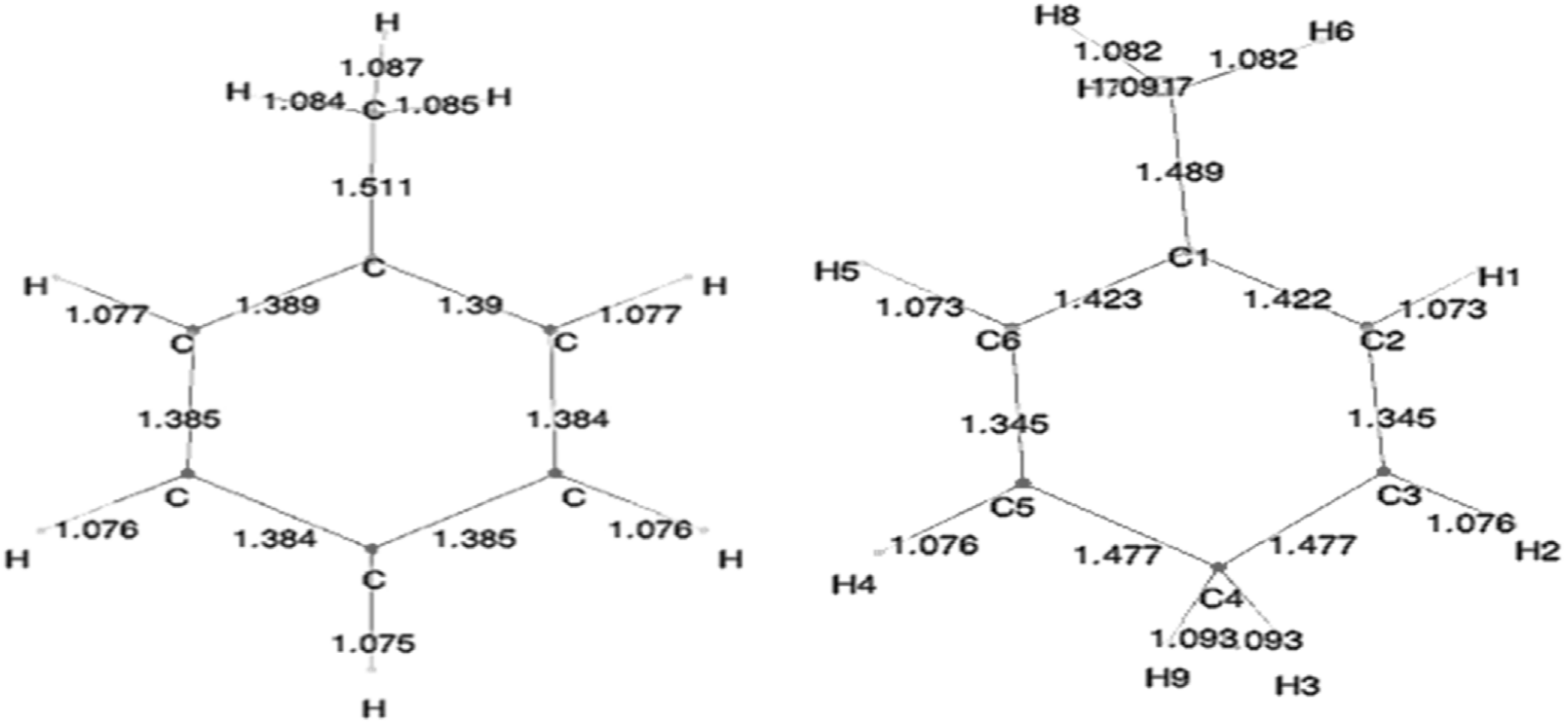
SCF-optimized bond lengths of toluene and p-toluenium (Å).

Geometry optimization calculations for each of the molecules were carried out at each of the levels of theory. As expected, in the presence of HC, the bond lengths of certain carbon–carbon bonds in the aromatic ring are expected to be impacted. Figure 3 shows the calculated bond lengths for benzene and benzenium, and Figure 4 shows those for toluene and toluenium.

The optimum benzene geometric parameters are in close agreement with the experiment. In the presence of HC, the bond lengths of the C1–C6 and C3–C4 bonds are shortened whereas C1–C2, C5–C6, C5–C4, and C3–C2 are lengthened. As seen in Figure 4, which shows the bond lengths in benzenium, all of the carbon–carbon bonds are near 1.39 Å, somewhat shorter than the values of the standard value for an aromatic carbon–carbon bond length (1.47 Å). Additionally, all of the carbon–carbon bond lengths lie near the standard aromatic bond length of 1.39 Å in the toluene molecule as shown in Figure 4, indicative of the presence of HC.

. In addition, the aliphatic carbon–carbon bond length (C7–C1, of 1.511 Å in toluene, shorter that the standard value of 1.54 Å) indicates the presence of HC. Furthermore, the C7–C1 bond length in toluene is calculated as 1.511 Å, but shortens to 1.489 Å in toluenium. Energies of the toluenium isomers and their respective proton affinities are reported in Table 4.

**TABLE 4:** Total energies, HOMOs, LUMOs, and proton affinities for toluene and the toluenium isomers.

	Toluene	o-Toluenium	m-Toluenium	p-Toluenium	ipso-Toluenium
Energy (a.u.)					
SCF	−269.8000	−270.1214	−270.1135	−270.1243	−270.1099
HOMO	−0.3235	−0.541	−0.542	−0.554	−0.553
LUMO	0.1362	−0.166	−0.173	−0.160	−0.174
CISD	−270.5619	−270.9219	−270.9149	−270.9245	−270.9109
DFT	−271.6063	−271.9496	−271.9425	−271.9516	−271.9371
CCSD(T)	−270.8216	−271.0991	−271.0928	−271.1018	−271.0886
Proton affinity of toluene Kcal/mol					
SCF	-	201.7	196.37	203.5	194.5
CISD	-	225.9	221.5	227.5	219.0
DFT	-	215.4	211.0	216.7	207.6
CCSD(T)	-	174.1	170.2	175.8	167.5

Calculations of electric dipole polarizabilities of benzene, benzenium, toluene, and all toluenium isomers at the optimized geometries were carried out at different levels of theory [SCF, DFT (B3LYP), CISD, and CCSD(T)]. As is shown, the SCF polarizability of benzene is calculated to be 58.45 a.u. compared to that of benzenium, which is smaller by 1.04 a.u. Also shown are the electric dipole polarizabilities calculated at the CISD level of theory, with the benzenium value smaller by 0.8 a.u. The electric dipole polarizability of toluene is 70.36 a.u., compared to that of toluenium, which is smaller by 0.48 a.u. As shown, the calculated values for both benzene and toluenium are all lower than the experimentally determined values (NIST Chemistry WebBook). Furthermore, the differences in polarizabilities between the neutral and protonated molecules indicate a tightening of the electron clouds for the latter, with the effect more pronounced for toluene.

The different structural isomers of toluenium were examined following geometry optimization calculations on each of the isomers. Examination of the methyl group at different positions (ortho, meta, para, or ipso Figure 2) is important because in determining how the position of the methyl group affects HC. The results are presented in Table 5 and Table 6.

**TABLE 5:** Average bond lengths (Å), polarizabilities (a.u.), and HCH angles of benzene and benzenium at different levels of theory. Experimental values are shown in parentheses.

Benzene	DFT	CISD	SCF	CCSD(T)
RCH	1.08	1.08	1.08	1.09 (1.08)
RCC	1.39	1.39	1.39	1.4 (1.39)
Polarizability	59.957	57.944	58.455	57.593
Benzenium				
RCH	1.08	1.08	1.07	1.08 (1.08)
RCC	1.37	1.36	1.35	1.37 (1.36)
Polarizability	59.111	57.853	57.411	58.892
Θ(HCH)	100°	103°	103°	102°

**TABLE 6:** Average bond lengths (Å), polarizabilities (a.u.), and HCH angles of toluene and toluenium isomers at different levels of theory. Experimental values are shown in parentheses.

Toluene	DFT	CISD	SCF	CCSD(T)
RCH	1.08	1.08	1.08	1.09 (1.08)
RCC	1.39	1.39	1.38	1.4 (1.39)
Polarizability	73.232	69.750	70.358	69.442
m-Toluenium	DFT	CISD	SCF	CCSD(T)
RCH	1.08	1.08	1.08	1.09
RCC	1.37	1.36	1.35	1.37
Polarizability	70.502	67.873	67.858	65.598
o-Toluenium	DFT	CISD	SCF	CCSD(T)
RCH	1.08	1.08	1.07	1.08
RCC	1.36	1.35	1.34	1.37
Polarizability	72.970	70.421	69.681	72.106
p-Toluenium	DFT	CISD	SCF	CCSD(T)
RCH	1.08	1.08	1.07	1.07
RCC	1.36	1.35	1.35	1.36
Polarizability	71.798	69.036	68.590	70.257
ipso-Toluenium	DFT	CISD	SCF	CCSD(T)
RCH	1.08	1.08	1.07	1.08
RCC	1.37	1.36	1.34	1.36
Polarizability	70.024	67.392	67.154	68.658

As can be seen in Table 4, the protonation energies are relatively close to one another. That is, there are no major differences between the total energies, HOMOs, and LUMOs of o-, m-, and p-toluenium. This indicates that the methyl group position in the toluenium ion has a small impact on the strength of HC.

**FIGURE 5:**
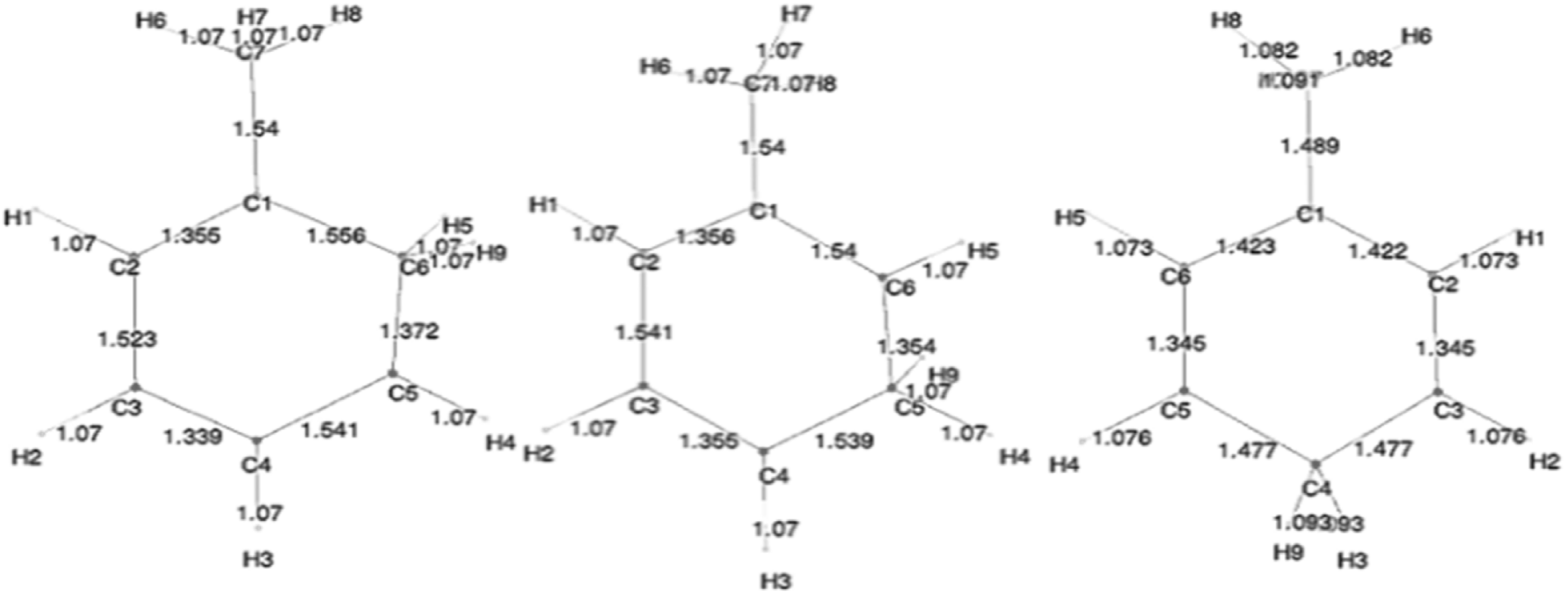
SCF-optimized bond lengths of o-, m-, and p-toluenium (Å).

Figure 5 shows the bond lengths in the isomers of toluenium. The bond lengths in each molecule are similar to one other with the p-toluenium showing slightly shorter bond lengths, indicating that the most stable isomer is the para form.

The mass spectrometric study of Kuck et al. (1985) on benzenium and toluenium syntheses revealed all of the structures shown in Figure 1 and Figure 2. Their results are consistent with the present calculated ones. Table 7 shows the Mulliken population analyses for benzene, benzenium, toluenium, and p-toluenium. Table 8 reports overlap populations for benzene, and Table 9 shows overlap populations for toluene and the four toluenium isomers.

**TABLE 7:** Comparison of Mulliken populations analyses as net charges for benzene, benzenium, toluene, and p-toluenium at the SCF and CISD levels of theory.

	Benzene	Benzenium	Toluene	p-Toluenium
SCF	C −0.099	C3 -0.113	C3 -0.076	C3 0.002
	C −0.099	C4 0.076	C1 -0.106	C1 -0.096
	C −0.099	C5 -0.113	C2 -0.114	C2 -0.014
	C −0.099	C2 -0.011	C6 -0.110	C6 -0.152
	C −0.099	C1 -0.163	C5 -0.076	C5 -0.014
	C −0.099	C6 -0.011	C4 -0.113	C4 -0.096
	H 0.099	H3 0.167	H1 0.096	H1 0.151
	H 0.099	H4 0.196	H2 0.086	H2 0.182
	H 0.099	H5 0.167	H3 0.086	H3 0.204
	H 0.099	H2 0.190	H5 0.097	H5 0.182
	H 0.099	H1 0.213	H4 0.095	H4 0.151
	H 0.099	H6 0.190	C7 -0.173	C7 -0.193
		H7 0.213	H6 0.112	H6 0.154
			H7 0.099	H7 0.181
			H8 0.096	H8 0.155
				H9 0.204
CISD	C −0.100	C3 -0.072	C3 -0.082	C3 -0.026
	C −0.100	C4 0.034	C1 -0.097	C1 -0.058
	C −0.100	C5 -0.072	C2 -0.107	C2 -0.029
	C −0.100	C2 -0.027	C6 -0.097	C6 -0.178
	C −0.100	C1 -0.190	C5 -0.082	C5 -0.029
	C −0.100	C6 -0.027	C4 -0.106	C4 -0.058
	H 0.100	H3 0.168	H1 0.095	H1 0.152
	H 0.100	H4 0.193	H2 0.085	H2 0.181
	H 0.100	H5 0.168	H3 0.085	H3 0.214
	H 0.100	H2 0.188	H5 0.095	H5 0.181
	H 0.100	H1 0.224	H4 0.094	H4 0.152
	H 0.100	H6 0.188	C7 -0.210	C7 -0.224
		H7 0.224	H6 0.119	H7 0.160
			H7 0.104	H6 0.187
			H8 0.104	H8 0.160
				H9 0.214

**TABLE 8:** Overlap populations for benzene and benzenium.

	Benzene	Benzenium
C1–C2	0.514	0.391
C1–C6	0.515	0.589
C6–C5	0.514	0.389
C1–H1	0.434	0.434
C6–H5	0.038	0.030
C2–H2	0.434	0.425
C5–H7	0.038	0.395

**TABLE 9:** Overlap populations for toluene and toluenium isomers.

	Toluene	o-TH^+^	m-TH^+^	p-TH^+^	ipso-TH^+^
C1–C2	0.585	0.519	0.576	0.386	0.251
C2–C3	0.436	0.417	0.421	0.573	0.409
C3–C4	0.594	0.577	0.528	0.370	0.526
C4–C5	0.427	0.352	0.464	0.370	0.372
C5–C6	0.599	0.338	0.388	0.549	0.571
C6–C1	0.419	0.421	0.351	0.385	0.279
C3–C7	0.431	0.025	0.029	0.025	0.027
C7–H7	0.0003	0.0001	0.00001	0.0001	0.0004
C2–H1	0.037	0.434	0.439	0.446	0.423
C3–H2	0.039	0.431	0.426	0.424	0.431
C4–H3	0.005	0.432	0.435	0.401	0.421
C5–H9	-	0.266	-	-	-
C6–H5	0.004	0.428	0.389	0.446	0.429
C6–H9	-	-	0.274	-	-
C4–H9	-	-	-	0.398	-
C1–H9	-	-	-	-	0.037

The data revealed that, as the proton is added to the benzene ring, the overlap population decreases in that bond. For instance, all of the bond lengths in benzene are equal with overlap populations consistently 0.514–0.515. These are in contrast to the values of 0.389–0.391 in benzenium. This indicates that the addition of a proton contributes to the aromatic conjugation. In addition, HC affects the overlap population by increasing the strength of the π-bond and decreasing that of the σ-bond. It is well known that HC is present in benzenium and absent in benzene. With that fact, looking at Table 9, it is seen that the π-bonds in benzene are stronger compared to the σ-bonds in benzenium. That is, the C1–C2 π-bond in benzene is 0.514, in contrast to the σ-bond in benzenium, which is 0.391. While it is expected that σ-bonds should be stronger than π-bonds, due to the presence of HC, this is reversed as seen in benzene and benzenium. The same trend is seen for toluene and toluenium isomers in terms of overlap populations (the results in Table 9 are defined with reference to Figure 2 with the exception that in toluene, C1 is C3 and C4 is C6.). In this instance, it is seen that the presence of HC bonding range is estimated to be 0.370–0.398.

As Mulliken and Ermler noted out in their analyses of other *ab intio* studies of six-membered ring molecules, all indicate that the structures have the proton attached to one carbon atom to form a CH_2_ group perpendicular to the plane of the ring (Mulliken and Ermler, 1981). These earlier studies, all at the SCF level of theory, consistently predict proton affinities near 190 kcal/mol, within 10 kcal/mol of experimental values. The present correlated calculations are consistent with these SCF values, in keeping with the fact that all of the neutral and protonated molecules have the same numbers of electrons and the expectation that, in general, electron correlation does strongly impact proton affinities (Chandra and Goursot, 1996).

The Mulliken population analysis procedure was chosen to further analyze all of the SCF, CISD, CCSD(T), and DFT computational results beyond the energetics and other properties. Although it is implemented in Gaussian (Frisch et al., 2016) and nearly all currently available quantum chemistry software suites, Mulliken’s method has well-known shortcomings, especially revealed in his so-called one-half overlap assignment (Mulliken and Ermler, 1977). As was carried out in our earlier calculations on benzenium (Ermler et al., 1976) and p-toluenium (Ermler and Mulliken, 1978), this work compares the results of population analyses described with the same GTO basis set and within the same level of theory, thereby providing a means by which changes in gross and overlap populations from one molecule or isomer to another can be compared in the same context.

The Mulliken population analysis method has been revisited by a host of researchers who have refined it to address its embedded constraints. Noteworthy are the development of natural bond orbitals (NBOs) by (Reed et al. (1985); Reed et al. (1988)), the atoms in molecules (AIMs) approach of Politzer et al. (1970) and Bader (1985), Roby’s method based on electron density operators and projection operators (Roby, 1974), and the one based on electron localization functions (ELFs) (Silvi and Savin, 1994). In terms of the concept of hyperconjugation, the NBO method is especially compelling and can now be implemented in the context of existing software suites (Glendening et al., 2021).

There are also a number of the methods that do not deal with populations, but interpret electronic structure calculations through analyses of the molecular energy and total wave function. Two examples are the energy decomposition analysis (EDA) method (Mo et al., 2000) and a definition of aromaticity indices (Jug et al., 1991). While the analysis of molecular electronic structure calculations reported herein is based on geometric changes, protonation energies, polarizabilities, and Mulliken populations, future work that incorporates one or more of the aforementioned approaches can possibly provide additional insight into the nature of aromatic hyperconjugation.

## 4 Conclusion

This investigation into the effects of aromatic hyperconjugation in benzene, benzenium, toluene, and its isomers through high-level quantum chemical calculations shows good agreement with the available experimental data. The presence of HC in these molecules is evident from the observed shortening of carbon–carbon bond lengths in the aromatic ring and the diminishing of polarizabilities in the protonated forms. This phenomenon contributes to the stabilization of these molecules and can play a crucial role in determining their structural and electronic properties. The proton affinities of benzene and toluene calculated in this study are in close agreement with experimental values, further supporting the accuracy of the calculations and the role of HC in these systems.

Additionally, the investigation of all of the toluenium isomers reveals that the position of the methyl group has a minor impact on overall strength of the HC. However, the most stable isomer was found to be the para form, consistent with the influence of HC on the molecular stability and structure. The analysis of Mulliken population data further substantiates the presence of HC in the molecules, showing that the addition of a proton contributes to aromatic conjugation and strengthens π-bonds while weakening σ-bonds. This emphasizes the important role of HC in modifying electronic distributions and bonding patterns within these aromatic systems.

Overall, this investigation shed some light on the significance of hyperconjugation in aromatic molecules, highlighting its influence on structural stability, reactivity, and electronic properties. The implications of these results can be extended to various areas, including catalysis, reaction mechanisms, and the stability of aromatic species.

## Data Availability

The datasets presented in this article are not readily available. Requests to access the datasets should be directed to walter.ermler@utsa.edu.
